# A Feasibility Study Assessing Acceptability and Supply Issues of Distributing LPG Cookstoves and Gas Cylinders to Pregnant Women Living in Rural Bangladesh for Poriborton: The CHANge Trial

**DOI:** 10.3390/ijerph17030848

**Published:** 2020-01-29

**Authors:** Camille Raynes-Greenow, Sajia Islam, Jasmin Khan, Fariha Tasnim, Monjura Khatun Nisha, Jonathan Thornburg, Sk Masum Billah, Ashraful Alam

**Affiliations:** 1 The University of Sydney, Sydney School of Public Health, Edward Ford Building (A27), Camperdown, NSW 2006, Australia; mnis2091@uni.sydney.edu.au (M.K.N.); neeloy.alam@sydney.edu.au (A.A.); 2 Maternal and Child Health Division, icddr,b, Mohakhali, Dhaka 1212, Bangladesh; 3 RTI International, 3040 Cornwallis Road, Research Triangle Park, NC 27707, USA

**Keywords:** Feasibility, household air pollution, LPG cookstoves, perinatal mortality, Bangladesh

## Abstract

Our aim was to develop a protocol for a cluster randomised controlled trial to assess the impact of liquid petroleum gas (LPG) cooking compared to usual cooking on perinatal mortality in pregnant women in rural Bangladesh. We, therefore, aimed to assess the feasibility of the planned trial and the barriers/facilitators of distributing LPG to rural households. We conducted a feasibility study in rural Bangladesh using an iterative design. We included pregnant women, their families, and local LPG stakeholders. We distributed LPG to households for 3 months (3 cylinders) and assessed process issues, acceptability, and cooking/food behaviours. We interviewed LPG stakeholders, and conducted focus groups and in-depth interviews with the users. The initial distribution and uptake of LPG were hampered by process issues, most of these were due to the non-established supply chain in the study area. LPG cooking was very acceptable and all users reported a preference for continued use, fuel-sparing was heavily practiced. Safety concerns were an initial issue. LPG stakeholders reported that LPG demand differed by season. This study demonstrated the feasibility of our planned trial and the need for safety messages. These results are relevant beyond our trial, including for programs of LPG fuel promotion.

## 1. Introduction

Household air pollution is one of the major contributors to the global burden of morbidity and mortality [1], and although women and children experience the greatest exposure burden, the impact on perinatal mortality (stillbirth >28 weeks and early neonatal death ≤ 7 days after birth) has not been accurately defined [2]. In South Asia, both the reliance on polluting fuels that cause household air pollution and perinatal mortality is high, and this, coupled with the accumulating evidence of the effect of exposure to household air pollution and perinatal outcomes, suggest that further research is needed [2,3].

A 2014 systematic review and meta-analysis of stillbirth with polluting fuels reported a summary odds effect of 1.29 (95% CI: 1.18, 1.41) [4], which is slightly lower than the previous estimates from an earlier systematic review (OR: 1.51, 95% CI: 1.23, 1.85) [2]. In both reviews, the same four studies were included [5,6,7,8] with the exception of an additional study in the more recent review [9]. The quality of the included studies in the reviews was rated as weak due to the mostly retrospective designs with a high risk of information and selection bias, also several of the studies did not account for important risk factors in the analysis, and the more recent review noted that there was evidence of publication bias. The population attributable risk estimate reported in the first review was 26% for stillbirth, but this was using a prevalence of solid fuel use at 70%, our data in rural Bangladesh suggest a prevalence of 98%. This is based on a recent randomised controlled trial of approximately >30,000 rural pregnant women in Bangladesh [10]. Given the high exposure, the high burden of perinatal mortality and the poor quality of the evidence there is a need for a high-quality trial that will accurately assess the effect of cooking fuels on pregnancy.

We, therefore, designed a community-based cluster randomised controlled trial to assess the impact of liquid petroleum gas (LPG) compared to usual cooking (predominantly traditional biomass fuel stove) on perinatal mortality in pregnant women in rural Bangladesh (Poriborton: The CHANge trial, ACTRN12618001214224), the planned sample size was ~ 5000 pregnant women. However, before the design of the trial was finalised, we wanted to assess the feasibility of conducting such a large-scale trial. We used a broad definition of feasibility in that our methods were iterative, formative and adaptive [11]. This study although contained some components of our planned trial and in such was a pilot study it was not a mini version of the planned trial and did not include a control arm and was not randomised as these were issues that did not require evaluation. The major barrier to conducting a large trial was distribution of LPG cylinders to a rural area with limited LPG use, and how we could establish a supply chain sufficient to ensure continuous LPG supply to be efficacious for a trial. Therefore, the main aim of this study was to; assess barriers and facilitators of distributing LPG cylinders to pregnant women, assess cooking behaviours and acceptance of cooking with LPG and whether women would accept wearing a device to measure their personal air pollution exposure in rural households in Bangladesh.

## 2. Materials and Methods

We conducted a feasibility study of LPG stove and cylinder distribution. The study was conducted in Basail sub-district which comprises ~ 160,000 people and is located 110 km north of the capital Dhaka. We purposively selected three unions (the lowest administrative unit in rural areas, each with ~ 25,000 people) in Basail, namely Kanchanpur, Kashil, and Kawajani for the study.

The study population included three groups of participants; (1) pregnant women (gestational age between 12 to 20 weeks), residing in the study area for the study period, who were not currently using LPG stoves in their home, but were willing to use LPG for household cooking up to 3 months while pregnant, (2) the family members of the enrolled pregnant women (husband, and or mother/mother-in-law), and (3) stakeholders who had roles in distributing LPG (suppliers, retailers, and transport workers) in the region. Exclusions included women who were not able to complete the stove trial for the whole study period, either due to moving out of the study area or gestational age was >6 months or were already using LPG (this final group was included in a second study).

We used different methods for our three different study groups. Our first group, pregnant women, were identified through a door-to-door census of married women residing in the study site. The listing criteria were women of reproductive age, date of last menstrual period, gestational age, stove types and willingness to stay at the current address till the end of their pregnancy. For this group, we (1) distributed LPG stoves with a single cylinder and two replacement cylinders per household for a period of gas supply for approximately three months, (2) used structured questionnaires at baseline, (3) semi-structured questionnaires at approximately 8 intervals throughout the intervention period, (4) measured personal particulate matter (PM2.5) exposure using the RTI MicroPEM™ over 24 h at baseline (pre-LPG installation) and during the LPG installation period in a subset of women (n = 30), and (5) in-depth interviews approximately two months after the gas supply was stopped in a sub-set of women (n = 12).

Our second group consisted of family members of enrolled pregnant women, data were collected using (1) semi-structured interviews (of female family members) at baseline (n = 12), and focus groups (of male family members) at end line (3 groups, n = 20). Interviews took place at the participant’s home, or wherever was most convenient to them, and focus groups occurred in a central location.

For our stakeholder group, we conducted (1) semi-structured interviews throughout the study period to assess their roles in LPG distribution and challenges and facilitating factors of distributing LPG stoves and cylinders. These were conducted in a place that was convenient to them such as near their shop or delivery point and were held sometime between the 5th and 10th week of the intervention period.

Two trained qualitative researchers conducted all interviews and focus groups which were based on the semi-structured interview guide of a previous data collection [12]. All interviews and focus groups were recorded using digital audio recorders. Interviewers and note-takers took additional notes of the key terms, the environment in the interviews or focus group, and significant non-verbal communications. Audio recording of all interviews and groups discussions were transcribed verbatim into Bangla. Transcripts were expanded by adding the additional notes that were taken by the note-taker and interviewer. We checked a random sample of the transcripts against audio recording to ensure the quality of transcription.

**Figure 1:**
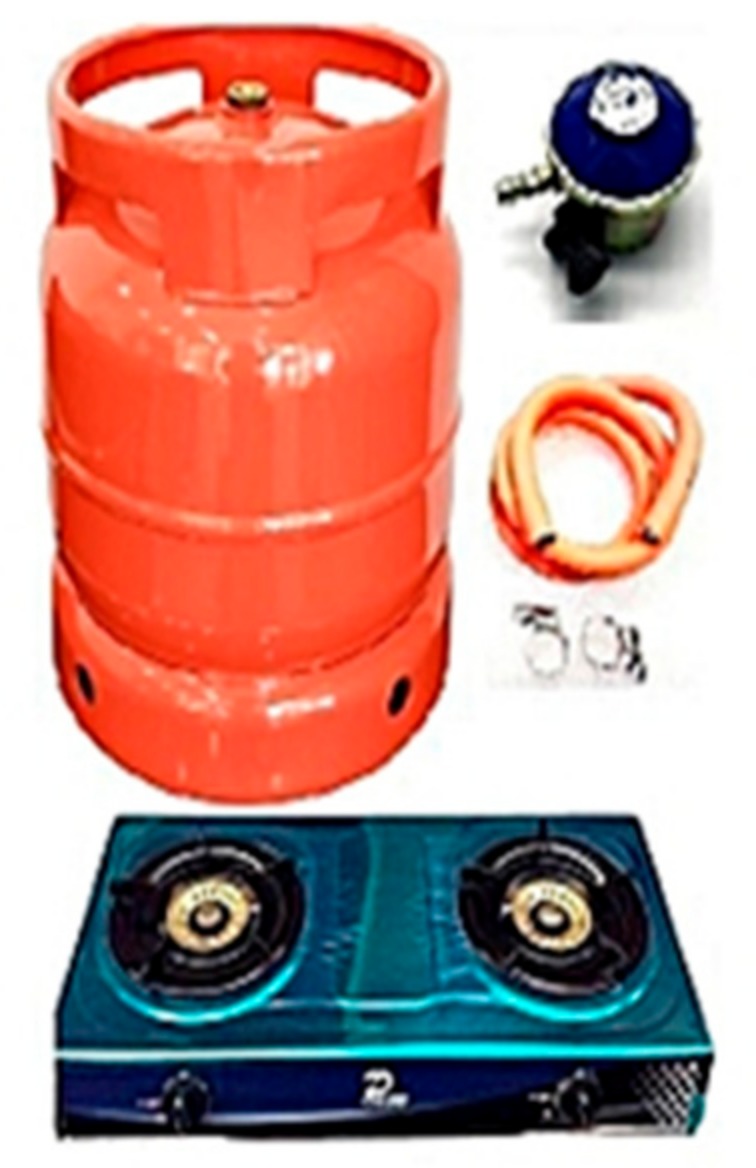
Stove, cylinder, hose, and regulator.

LPG stoves and cylinders were installed inside the home of the pregnant women, in a position of her choice, usually in an elevated position near a window (if possible) for ventilation. The stoves were locally made and available in the markets of the study site. They were small two burner stoves and were selected due to their low cost and availability in the area, they met the quality control of the national standard and testing institute and cost USD$13 (Figure 1). Cylinder size was 12 kg, the cost per cylinder with gas was USD32 (Cylinder deposit was USD16, and LPG USD16).

We followed all manufacturers’ recommendations about LPG stove placement and adhered to all safety regulations. We also provided safety information and operating demonstrations to all household members. At the conclusion of the study, both the cylinder and the stove were donated to the study participants. We used an iterative process for the distribution of the LPG in that if new evidence of problems or issues arose from any participant including stakeholders, we adapted our processes to overcome these and improve our cylinder distribution. We further collected anecdotal information throughout the study to troubleshoot any issues that arose including issues with stove use, and supply. This was an a priori decision to help improve the feasibility of LPG distribution.

### 2.1. Data Collection

Acceptability and use of LPG for cooking, cooking behaviours, time spent cooking, any other food and/or cooking issues (such as food taste, and learning how to cook with gas), fuel use, process issues with supply and set-up of the stoves, was collected as times described above. A kitchen observation checklist, detailed field notes, and where appropriate photographs in situ of the LPG stoves were also collected. We used an abbreviated version of the pregnant women’s questionnaire from the Bangladesh Demographic and Household Survey [13], and a very short topic list to guide the interviews with LPG stakeholders. Focus group discussions and in-depth interviews explored the same topics as the stakeholders with the addition of some questions about health perceptions attributed to stove use. The subset of 30 women in the personal exposure study were asked to wear the MicroPEM on a custom-made bag with a strap around the neck that kept the device on their chest at all times except while sleeping or bathing. A 3-axis accelerometer with 0.1 g sensitivity embedded in the MicroPEM detected the frequency and intensity of movement used to assess wearing compliance, defined as the percentage of time the unit was worn in compliance with the study protocol [14].

### 2.2. Sample Size

Sample size (N = 50) was based on pragmatic factors, and not quantitative factors, as is acceptable for feasibility studies, as the outcome was not the efficacy of the intervention [11]. Specifically, we were sampling based on budget, as we were conducting a study of feasibility (i.e., “can it work?”) we wanted to recruit as many women into the study as possible. Therefore, the number of participants was determined by the cost of the LPG (cylinders, stoves, and distribution) and research costs (staff). We wanted to maximise the number of women into the study to give us the largest sample and hence the broadest description of all domains of feasibility that were yet unknown.

### 2.3. Data Analysis

All data were collected in Bangla. In-depth interviews and focus group discussions were coded by the local experienced qualitative research team, overseen by the senior investigators. Codes were generated using Atlas.ti based on the a priori core analysis themes, that answered our specific objectives (deductive). However, we also used inductive coding as new themes emerged. Codes were summarised and agreed upon by the research team, then triangulated with the quantitative data (where appropriate) and any field notes. The final analysis was synthesised by the team through discussion. Themes were discussed in the team and summaries of findings were translated into English, and discussed between the team to ensure interpretation matched the data. The qualitative data was used to augment the quantitative data. We calculated frequencies with percentages for the selected variables to describe participants’ socio-demographic characteristics, household structure, cooking practice, and stove usage.

The MicroPEM software calculates wearing compliance. The MicroPEM is worn if the rolling standard deviation in the composite x-y-z acceleration over a 5-min window exceeded 0.02. Field staff also collected process issues and data on women’s experiences of using the device.

### 2.4. Ethics Approval

All participants provided informed consent before participation and were asked to report any injuries or incidents associated with LPG use. This study was approved by the Ethics Review Board of icddr,b (formerly known as the International Centre for Diarrhoeal Disease Research, Bangladesh), approval no. PR-15126.

## 3. Results

Using a household pregnancy surveillance survey method, we recruited 50 pregnant women into the study, and their family members (mothers/mothers-in-law, husbands). We held three focus groups of men with between 6–8 per group, and interviewed 12 stakeholders (9 shopkeepers, 2 drivers, and 1 supplier), and interviewed 12 enrolled women. Thirty women participated in the Micro-PEM sub-study, in this paper we present the compliance results, the exposure data presented elsewhere.

All women who were approached were willing to participate, although many women did not meet our eligibility criteria (either gestational age was too advanced or were current users of LPG n = 337). Participating women were generally not engaged in paid work (n = 44, ~ 90%), and most had at least primary school education (n = 47), there were seven women who had higher than secondary education (14.3%) (Table 1). All participating women were pregnant (up 22 weeks gestation at enrolment), and almost half had a previous pregnancy (n = 24), eight women reported a previous pregnancy loss. Women were aged between 15 years and 37 years. Participating women indicated that they were the main household cook however there was some task sharing with other female household members. The main cooking location (for traditional cooking) was in a separate structure that was used as a kitchen, and the construction of the kitchen varied from a roof with full wall by over half of the participants to cooking in the open outdoors, used by two participants. In the 27 households who cooked in a kitchen with a roof and a full wall, the majority were well ventilated (i.e., cross-ventilation with door and or windows (15)). The primary stove for all households was a traditional clay-made stove, and there was a sole reliance on biomass fuel (wood, crop remnants, cow dung etc.) (Table 1). All households had not cooked or used LPG previously, but all were aware of LPG, and their perception was that it was superior cooking compared to traditional stoves.

### 3.1. LPG Delivery Process and Stakeholder Issues

The initial distribution of stoves and LPG cylinders was delayed through an inability to identify a local distributor, as there was no established LPG cylinder shop or vendor in the study site. We, therefore, identified vendors who had some ad hoc experience with distributing LPG cylinders, and whose shop was central and large enough to accommodate at least ten cylinders. Negotiation with vendors, and drivers was considerable, the main issues were around cost and delivery of the stove and the cylinders. Supply of subsequent cylinders was also an issue for negotiation, as we required a no-gap supply. We also needed to negotiate the contact system between our participants and the vendors for re-supply of the two subsequent cylinders, as this had not been previously established. Specific issues included; how this would occur and how much notice was needed to ensure that a cylinder would be available and delivered quickly. Most of these issues arose due to the very low penetration of LPG into the area, which meant that there were not large numbers of cylinders available or a supply system established. Eventually, we arranged an initial supply of LPG stoves and cylinders to the identified shops. These shops were centrally located for the participants. All participating women were given an enrolment card with the address of the selected shop. The vendors were instructed to only provide the stove and, or cylinder if the woman or her representative could show our enrolment card. We provided the stoves and cylinders at no cost to the participants. The vendors were paid the equivalent of USD~25 cents for storing each cylinder which encouraged them to provide the replacement cylinder on time.

Low penetration of LPG into the area was highlighted by shopkeepers. These participants reported that they worked as door-to-door sales representatives to promote LPG and influence households to adopt, and also communicated with households of the benefits of LPG use compared to traditional fuel cooking. They claimed to have some success with increasing LPG uptake. Similar to shopkeepers, a supplier also reported motivating people to adopt LPG:

“I have been running my business since 1997. During the early stages of my business, it took me nearly 8 months to sell only 125 gas cylinders. I could manage only a few customers in Tangail after motivating relatively wealthier families to adopt gas stoves for cooking through several home visits. Back then people did not understand the benefits of gas cooking. Despite their sufferings from smoke, they used to prefer conventional clay stoves for cooking. I used to supply gas stoves to households on credit during those days.”

(Supplier)

Suppliers and shopkeepers all reported differences in demand for LPG according to season, and special occasions such as Ramadan and Eid. For instance, a store owner of LPG stoves said:

“The demand for gas is more in the rainy season when the wood, straw (solid fuels) remain wet most of the times. Gas demand is also more during Ramadan period.”

(Store owner)

Variable demand caused supply problems. Both suppliers and shopkeepers discussed issues with ensuring sufficient gas cylinder availability when there were issues further up the supply chain. They also reported issues in the availability of the actual cylinders by the gas companies who imported the cylinders who could not meet a high demand.

Delivery issues highlighted by drivers included poor roads, and flooding that prevented delivery. A van driver described carrying cylinders on his head when the van could not access a village. Another van driver reported similar issues and if van access to the shop was not possible, he would hire a motorcycle, or auto rickshaw (colloquially referred to as a ‘CNG’) to transport the products to the shop. The main issue impeding delivery were adverse road conditions mostly due to construction, or road repair. Another issue reported from drivers was theft from their trucks. Some roads were at higher risk of theft and where possible drivers would take an alternative route, or avoided stopping or slowing the vehicle, and preferred to have a helper in the van who could watch for theft. Drivers were also careful to keep their driving license and other papers up-to-date to avoid ‘police harassment’.

### 3.2. Household Experiences

**Figure 2:**
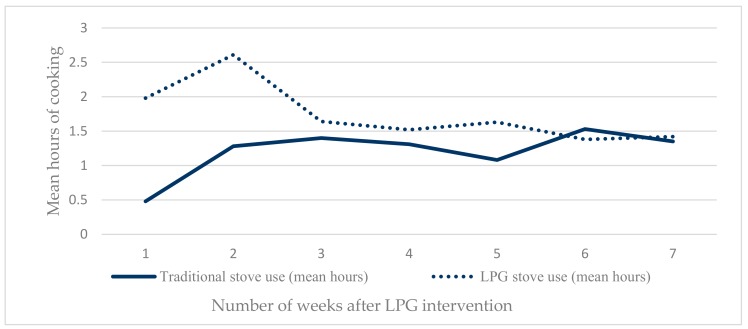
Mean hours of cooking in previous 24 h per visit by stove type.

All households were non-LPG users, with no previous experience of cooking with LPG before participation in our study. Most of the families set the cookstove on a few bricks stacked up or on a table in their home, which was different to the traditional stove placement in a separate kitchen structure. Although we asked all households to cook with LPG for the whole time, we did not provide any supporting behaviour change intervention. Participants were also aware that they were only receiving two LPG cylinders. Consequently, traditional solid-fuel cooking continued, with all households reporting that they continued to use their traditional clay stove at least twice a day and practiced fuel-saving with the LPG. This practice was evident when examining the stove use in hours by visit, which demonstrated participants’ high acceptance of LPG stoves during initial visits and then fuel-sparing behaviour in later visits (Figure 2). The mean frequency of LPG stove use was approximately four times (1.98 h vs. 0.48 h), and double (2.61 h vs. 1.28 h) the mean frequency of traditional stove use in visit 1 and visit 2 respectively. However, in subsequent visits the participants’ LPG use started to decrease and remained low, balanced by increased use of traditional clay stoves throughout the later visits (visits 3 to 8) (Figure 2). The main reason for LPG fuel saving was because it was highly valued. This strong preference was also identified as a barrier for the conduct of a trial, as households reported wanting to use LPG for as long as possible however the relatively high cost of the gas after the study period was a barrier to future use, and therefore not practice exclusive LPG use in the study period. In this regard a woman said:

“Because of the high cost of gas, we do not use the gas stove. We use traditional clay stove because of its low cost. If I had enough money then I would definitely use a gas cookstove, I would not use the traditional stove.”

(Woman M05)

There were some minor concerns about safety with using LPG that were expressed to us in the initial LPG set-up period. Families were unsure about how to use the stove and were worried about their safety. These issues were initially allayed during the set-up of the stove, as our staff demonstrated correct and safe use, and all available household members received safety advice and instructions. After set-up no participants reported safety issues as a barrier to using LPG. The suppliers had also mentioned that there was a general concern for safety in rural areas with using LPG cylinders (specific risks included connection to the stove and explosion risk) which they resolved during their sales conversations with prospective clients.

“People of this area used to be scared of gas cooking before. They were scared if there would be any fire or burst of cylinders. I used to advise them to buy a gas stove. First, a few people bought the gas stoves. Then a few more people were motivated to buy the gas stoves. Initially, I had to explain the benefits of gas cooking such as no smoke occurs from gas stoves, cooking with gas stoves allow to be cleaner. Thus, I was able to motivate people of this area to use the gas stove.”

(Shopkeeper)

Male household members generally considered that LPG cooking was safer than traditional stoves as the fire used for the stove required supervision, and that the traditional stove had to be covered after cooking, as the fire stays active and can be spread to the other fuel scattered around the burner. All female participants were interested in converting to LPG and continuing use after the study period. Reported advantages of using LPG, included no smoke, and reduced soot on clothes, pots and walls, no eye irritation, convenient to use and reduction in cooking times. For instance,

“While cooking with traditional stove, sometimes I cannot see anything because of too much smoke from it. I feel bad that time as in that situation I need to continue my cooking. But the gas stove is very easy to use. There is no smoke from it, no blowing air is required. I just can cook only sitting. There will be no problem with cooking if I sit relaxedly and chat with someone during cooking with the gas stove. But with the traditional stove this is not possible as to keep the stove on blowing smoke is required which causes darkness and eye irritation, thus causes breathlessness. Therefore, I like the gas stove.”

(Woman, M05)

Women also appreciated that they could cook inside the home and did not need to be sitting down at the time of cooking as (some) stoves were placed on a table. Connecting the cylinder was done mostly with the help of a male family member, although some women reported that when there was no one available the women would connect the cylinder and stove themselves. All households recalled the safety and set-up instructions from our project staff.

Men also reported differences to usual practice, with men assisting women with turning the stove off and on and lifting the pot on and off the burner and a few men also reported taking on the role of cooking with the LPG when their wife was visiting her parents’ home. Husbands also reported that their wives were very eager to continue cooking with the LPG cookstoves, as the experience was favourable. Women wanted to use the LPG as their regular fuel, and asked their husbands to replace it quickly when it was empty. Husbands thought that it was acceptable to collect a new cylinder when they went to the bazaar or market-place as part of their regular duties.

There were several malfunctions with the stoves (knobs breaking, or falling off were the main problems), which meant that they could not be used, these were repaired but there was some delay in the timeliness of the repair.

Other practical issues related to conducting a future trial were that a few families were conducting large house repairs or construction that made their cooking arrangements different to usual practice, such as not having a fixed cooking location. Also, several of the enrolled women left their LPG household to visit other family during the study period and or returned to her parents’ home for the birth. Seven households reported that they had shared the LPG with their neighbours.

The final barrier for LPG use relevant to conducting a trial was that the LPG stoves were too small for cooking for larger families, and for cooking large amounts such as during harvesting or reaping season and special occasions, and also for cooking special foods like puffed rice, or making a traditional cake during the winter. There were no injuries reported from stove use.

**Figure 3:**
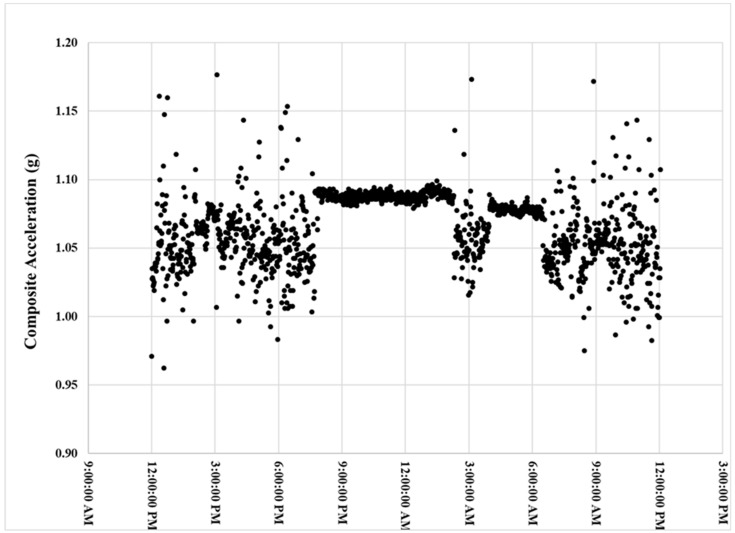
MicroPEM composite acceleration time series that illustrates the participant’s activity level and 89% compliance wearing the device according to the study protocol.

The women’s mean MicroPEM wearing compliance was 77% ± 6% (min–max: 49%–93%) and 69% ± 6% (min–max: 43%–89%) for baseline and LPG use phases, respectively. Figure 3 is an example accelerometer time series graph for a participant with 88% compliance. The participants did not report any negative or positive feedback from wearing the MicroPEM.

## 4. Discussion

We learned many valuable lessons and established the feasibility of conducting a large-scale trial of LPG for pregnant women in rural Bangladesh taking into account local issues and cultural cooking practices. We found that early identification of pregnant women was easily achieved through systematic household menstrual surveillance. The MicroPEM wearing compliance exceeded the minimum thresholds (40% to 60%) for representative exposure assessment established for other cohorts [14,15,16]. This suggests the full study will representatively measure exposure and associated impact on perinatal and neonatal health. Most importantly, the feasibility study established that households would readily adopt an LPG stove. The most attractive attributes of LPG adoption were low smoke emission, convenient use, cleanliness and reduced cooking time. Most importantly we were able to establish a distribution chain in a rural non-LPG established area. However, it also highlighted barriers that need to be overcome.

The supply and distribution chain were the most problematic for us. Some specific issues identified in this study were the delay in the LPG distribution (between participant enrolment and receiving the stove and cylinder), an inability to find a local distributor, problems with the supply of second and third cylinder due to limited space in the markets stalls and timing to meet expectations of no-gap supply of LPG. These supply and distribution barriers to LPG adoption were also echoed in previous studies conducted in Indonesia [17], Brazil [18] and Guatemala [19]. For us, this was partly a function of conducting the study in a small LPG market where LPG fuel use was very low, and where there was not an established supply chain sufficient for our needs. These issues were further exasperated for us as we were conducting a very small study, over a short time frame, on a small budget and did not have the purchasing power to negotiate or invest in establishing a supply chain. Although this study did not establish our supply chain, we used the experience of conducting the study to inform the discussions with our LPG contractor to establish a supply chain for our main trial. For our main trial, we have moved the study site to a different region, and have used the same process to identify local market vendors in geographically central locations for our intervention clusters. A previous study in Indonesia has demonstrated that preparing LPG retailers was beneficial to ensure the readiness of LPG supply during and after distribution activities, including the monitoring of LPG refilling [17]. For our main trial we have conducted training for the LPG vendors and keep in regular contact.

The finding that LPG demand differed by season and during festivals, has implications for LPG supply in conducting a trial, in which we will need to ensure sufficient supply in higher demand periods. The monsoon season occurs in Bangladesh between June and August, and we have considered this higher demand in this season for the supply chain in our main trial, by ensuring that each LPG stall holders/our distributors have sufficient cylinders to meet demand. An obvious solution would be to give two cylinders per household, and although we considered this it was not feasible due to the cost.

Most families reported continuing cooking with traditional clay stoves in order to save fuel, and this has been previously reported in rural India [20] and rural Ghana [21]. In a trial environment, we need to ensure that households adhere to the trial protocol and switch cooking fuels to LPG, so that the LPG intervention will be efficacious. We acknowledge that the poor adherence to LPG cooking in this study is a limitation, however it was never our intention as we were focused more on the process issues of LPG cylinder distribution. The main driver of the poor LPG use was ‘fuel saving’ due to the provision of only three cylinders. This will not be as issue in our main study as fuel will be provided throughout pregnancy. More importantly, based on extensive formative research of behavioural determinants (manuscript in preparation), we are supporting households with behaviour change communication to promote consistent use of LPG and are discussing options for micro-finance or other schemes that can support LPG purchasing in the post-study period.

Other than the process issues that we needed to solve, we also identified some practical issues that needed solving. The stoves we used in the feasibility study were problematic, with a small burner size, and knobs that frequently broke. We are not using these stoves in our trial. The new stoves accommodate a larger pot size that assists with some of the cooking issues and thus far we have not had a broken knob. In the behaviour change material, we suggest that women enlist the help from other household members or neighbours if they need to cook for a large gathering or a special recipe that the intervention stoves do not accommodate so they avoid exposure during these activities.

The behaviour change materials in our large trial also address the safety concerns about LPG cooking, which has been previously identified [17,21]. Safety mostly regarding the LPG cylinders is being included early in the trial so that it does not interfere with recruitment of the participants. Safety is a focus of our video, written material and face-to-face counselling to ensure that all families safely use LPG and are no longer fearful. As part of this, our intervention team are given intense training on LPG installation, connection, and troubleshooting all underpinned with safety measures. We are also holding regular community meetings in the study area to discuss the safety precautions.

Despite these barriers, most participants had a positive attitude towards using LPG stoves compared to the traditional clay cookstoves primarily because of the low smoke emission, convenient use, cleanliness and reduced time to cook. This is a critical factor as participants’ acceptability is crucial for conducting this trial.

## 5. Conclusions

This feasibility study suggests that a large LPG trial in rural Bangladesh is feasible if it considers the supply chain barriers, rural families’ needs, practices, and preferences. Overall, the recruitment process and exposure measurement over the study period were very good. Various behavioural change strategies are required to support women and their families’ cooking behaviours to adopt LPG cooking, and these lessons are worth considering when developing a new LPG program.

## Figures and Tables

**Table 1:** Background characteristics of the study participants.

Background Characteristics		N = 50 n (%)
Maternal age *	19 years or below	19 (38.8)
	20–29 years	24 (49.0)
	30 years or above	6 (12.2)
Women’s highest level of education *	None	2 (4.1)
	Primary	10 (20.4)
	Secondary	30 (61.2)
	Higher	7 (14.3)
Women’s employment status *	Employed	5 (10.2)
	Unemployed	44 (89.8)
Husband’s highest level of education *	None	5 (10.2)
	Primary	15 (30.6)
	Secondary	26 (53.1)
	Higher	3 (6.1)
Husband’s occupation *	Unemployed	1 (2.0)
	Unskilled labour	3 (6.1)
	Skilled labour	19 (38.8)
	Business	10 (20.4)
	Service	15 (30.6)
	Other	1 (2.0)
Number of household members *	2–4	19 (42.2)
	5–7	19 (42.2)
	>8	7 (15.6)
Number of rooms in household *	1	12 (24.5)
	2	21 (42.9)
	3	10 (20.4)
	>4	6 (12.2)
Roof material *	Tin	49 (100.0)
Wall material *	Tin	47 (95.9)
	Cement	2 (4.1)
Floor material *	Earth/sand	35 (70.4)
	Cement	14 (28.6)
Primary household cook by	Self	50 (100.0)
Kitchen location	Separate house used as kitchen	44 (88.0)
	In a separate room from sleeping	3 (6.0)
	In room used for sleeping and cooking	0 (0.0)
	Outdoors	3 (6.0)
Primary stove	Traditional clay-made stove	50 (100.0)
Stove used other than cooking	No	50 (100.0)
Fuel used **		
	Biomass (straw, grass, leaves, crop remnants, husks)	47 (94.0)
	Cow dung	47 (94.0)
	Wood	48 (96.0)
	Kerosene	0 (0.0)
	Coal	0 (0.0)
	LPG	0 (0.0)
	Electricity ***	2 (4.0)
	others	2 (4.0)
Smoking inside house	Yes	24 (48.0)
	No	26 (52.0)

* Data missing, ** Multiple responses, *** Rice cooker only.
